# POGLUT2 and POGLUT3: Two essential protein *O*-glucosyltransferases modifying EGF repeats in extracellular matrix proteins☆

**DOI:** 10.1016/j.bbagen.2026.130942

**Published:** 2026-04-08

**Authors:** Nicholas R. Kegley, Bernadette C. Holdener, Robert S. Haltiwanger

**Affiliations:** a Complex Carbohydrate Research Center, Department of Biochemistry and Molecular Biology, University of Georgia, Athens, GA, USA; b Department of Biochemistry and Cell Biology, Stony Brook University, Stony Brook, NY, United States of America

**Keywords:** Extracellular matrix, Fibrillin, Glycoprotein, Glycosyltransferase, Marfan syndrome, *O-*glucose, POGLUT2, POGLUT3

## Abstract

*O*-glycosylation of Notch epidermal growth factor-like (EGF) repeats has long been studied in the context of protein folding, secretion from cells, and protein function. Originally, the protein *O*-glucosyltransferase, POGLUT1, was thought to be the only enzyme adding an *O*-glucose modification to EGFs, specifically to a serine between cysteine one and two of a six cysteine containing EGF repeat. The POGLUT1 *O*-glucose can be elongated on Notch EGFs with xyloses by GXYLT1/2 and XXYLT1, forming a trisaccharide. Mouse knockouts of *Poglut1* are embryonic lethal with *Notch1*-related phenotypes. Recently, protein *O*-glucosyltransferases POGLUT2 and POGLUT3 were shown to add an *O*-glucose modification to a serine located between cysteine three and four, distinct from the POGLUT1 modification. This modification was first discovered on EGF11 of NOTCH1, then subsequently mapped on extracellular matrix proteins fibrillin-1 (FBN1), fibrillin-2 (FBN2), and latent transforming growth factor beta-binding protein 1 (LTBP1). *Poglut2/3* double knockout mice exhibit neonatal lethality like *Fbn1* or *Ltbp1* knockouts. In addition, *Poglut2/3* double knockout mice display syndactyly, similar to *Fbn2* knockouts. These studies also showed decreased secretion of FBN1 and 2 from fibroblasts and incorporation into the extracellular matrix. Site mapping established a putative POGLUT2/3 consensus sequence: C^3^-x-N-T-x-G-S-F/Y-x-C^4^. Alanine variants at conserved residues redefined consensus to: C^3^-x-x-x-x-x-S-x-x-C^4^. Marfan syndrome (MFS) is caused by FBN1 variants, and MFS variants in the POGLUT2/3 consensus display aberrant *O*-glucosylation. With the neonatal lethality of mouse *Poglut2/3* double knockouts and the changes in *O*-glucosylation caused by MFS variants, further examination of the effects of *O*-glucose on FBN1 function are needed.

## 1. *O*-linked glycosylation of epidermal growth factor-like repeats

In mammals, *O*-linked glycosylation refers to sugar modification of the hydroxyl functional group found on serine, threonine, or hydroxylysine side chains. Sugars can be galactose, *N*-acetylglucosamine (GlcNAc), *N*-acetylgalactosamine (GalNAc), mannose, fucose, and glucose [1–3]. These sugar modifications facilitate protein secretion from cells [4–8], enhance protein fold stability [7,9–11], and modulate the Notch signaling pathway [6,12–17]. Notch receptors initiate a signaling pathway that is required for development of all metazoans, and defects in the Notch pathway cause diseases like cancer (T cell acute lymphoblastic leukemia) and Alagille syndrome [18]. NOTCH1 is highly glycosylated on its epidermal growth factor-like (EGF) repeats (Fig. 1) with *O*-fucose, *O*-glucose, and *O*-GlcNAc [19]. EGFs are 40–50 amino acid domains defined by six conserved cysteines and three disulfide bonds [20–22].

In mammalian cells, *O*-glycosylation of EGFs is mediated by several glycosyl transferases (Fig. 1). The first *O*-glucose modification to be discovered on NOTCH1 was mediated by Protein *O*-glucosyltransferase 1 (POGLUT1) which utilizes the donor UDP-glucose to add an *O*-glucose to a serine (S) residue located between the first and second cysteine (C^1^ and C^2^) of the EGF consensus sequence: C^1^-x-S-x-P/A-C^2^ [27,28]. The *O*-glucose can be further elongated with two xylose sugars, with the first xylose added by either GXYLT1 or 2 [29], and the second by XXYLT1 [30]. *O*-fucose modification of NOTCH1 EGFs are mediated by Protein *O*-fucosyltransferase 1 (POFUT1) which utilizes GDP-fucose to add an *O*-fucose to an S or threonine (T) residues located within the consensus sequence C^2^-x-x-x-x-S/T-C^3^ [31]. The *O*-fucose can be further elongated with GlcNAc, galactose, and sialic acid [32]. Database searches with their respective consensus sequences yields over 100 potentially modified human proteins for POFUT1 [25], and over 50 for POGLUT1 [28], but the Notch receptors have more *O*-glucose and *O*-fucose sites than any other protein in the databases. Finally, the EGF-specific *O*-GlcNAc transferase (EOGT) utilizes UDP-GlcNAc to add an *O*-GlcNAc modification to S or T residues within the consensus C^5^-x-x-G-x-S/T-G-x-x-C^6^, which can also be elongated with galactose and sialic acid [33,34]. *O*-GlcNAc has been found on 15 different proteins through mass spectral-based analyses [35].

Without these sugar modifications the Notch receptors do not function properly. POGLUT1, POFUT1 and EOGT all reside in the ER where they only recognize and modify properly folded EGF repeats, suggesting that these enzymes serve as folding sensors for EGFs [2,8]. Consistent with this prediction, POGLUT1, POFUT1, and EOGT *O*-glycans stabilize the EGF fold and facilitate protein trafficking to the cell surface [9,10], which is essential for Notch function [6–8,35]. In addition, the *O*-fucose modification on EGF12 of NOTCH1 directly interacts with Notch ligands like Delta-like 4 (DLL4) and Jagged 1 (JAG1) on adjacent cells [12,31,36,37]. Eliminating the *O*-fucose site on EGF12 of mouse NOTCH1 or knocking out *Pofut1* (*Pofut1 KO*) in mice blocks NOTCH1-ligand interactions and results in embryonic lethality with embryonic defects similar to *Notch1* mutants [8,38–40]. Eliminating the *O*-fucose site on *Drosophila* Notch EGF12 prevents rescue of the Neurogenic and Lethality phenotype in *Notch* null *Drosophila* [41]. Similarly, *Poglut1 KOs* in mice and *Drosophila* (POGLUT1 is called Rumi in flies) result in Notch phenotypes [13,14]. In humans, mutations affecting these *O*-glycosyl-transferases cause cancer (POFUT1, POGLUT1), Dowling-Degos disease (POFUT1, POGLUT1), limb-girdle muscular dystrophy (POGLUT1), and Adams-Oliver syndrome (EOGT) [17,42–46].

## 2. Discovery of POGLUT2 and POGLUT3

POGLUT2 and POGLUT3 (POGLUT2/3), initially referred to as KDELC1 and KDELC2 [47], were originally identified as homologs of POGLUT1 due to their sequence similarity. However, they did not catalyze the transfer of a glucose from a UDP-glucose donor to an EGF repeat containing the consensus site for POGLUT1 [27]. Clues to their function was the identification by mass spectral analyses of a hexose on Notch EGF11 Serine (S435) located between C^3^ and C^4^ and also the presence of the hexose on Notch EGF11 S435 in a co-crystal structure of NOTCH1 and associated ligand DLL1 [37,48]. KDELC1/2 were shown to modify S435 with an *O*-glucose only when NOTCH1 EGF11was properly folded, while POGLUT1 could not modify S435 [6]. Conversely KDELC1/2 were unable to modify the POGLUT1 sites in EGF12 and 13 [6]. This *O*-glycosyltransferase activity led to the renaming of KDELC1/2 to POGLUT2/3. The selective ability of POGLUT2/3 to recognize and modify only folded EGF11 suggested that they are also folding sensors in the ER, similar to POGLUT1 and POFUT1. The redundancy of POGLUT2/3 was also discovered in this same study, where both *POGLUT2* and *POGLUT3* had to be knocked out in HEK293T cells to eliminate the *O*-glucose modification on NOTCH1 S435. Although mutating S435 to alanine on EGF11 had no effect on NOTCH1 signaling, when paired with an *O*-fucose variant on EGF12, activation of NOTCH1 by Delta-like 1 was reduced [6].

POGLUT2 and 3 are classified as members of the GT90 CAZy (carbohydrate active enzyme) family, which includes their homolog POGLUT1 [49]. Based on their sequence conservation [4,6,50], POGLUT2/3 are classified as GT-B glycosyltransferases, which are not dependent on metal ions for their activity. Because of their similarity to POGLUT1, both POGLUT2 and 3 are likely inverting glycosyltransferases, enzymes that invert the linkage of the sugar donor glycosidic bond when modifying an amino acid (in this context, going from an alpha linked glucose on UDP to beta linked *O*-glucose modified serine) [27,51–53]. POGLUT2 and 3 contain several structural features, including CAP10 glycosyltransferase domains, filamin domains (whose function has yet to be determined), and KDEL-like ER retention sequences [27,54]. In addition to their capacity to *O*-glucosylate EGF substrates, POGLUT1, 2, and 3 can also *O*-xylosylate EGFs *in vitro* when UDP-xylose is used as donor substrate [6,27]. UDP-glucose and UDP-xylose differ only by a single hydroxymethyl group, making it possible for UDP-xylose to be utilized as a donor substrate. However, UDP-xylose is used less efficiently by POGLUT1, 2, and 3, and this modification has not been found in protein isolated from cultured cells or mouse tissues [4,55].

POGLUT2 and 3 were initially thought to modify a strict consensus sequence of C^3^-x-N-T-x-G-S-F-x-C^4^; where the underlined S denotes the modified serine located between cysteines 3 and 4, and x denotes any amino acid other than cysteine (Fig. 1). This sequence was constructed by comparing POGLUT2/3 modified sites on Notch receptors [6]. Using this sequence to search protein databases identified Fibrillin 1 (FBN1), FBN2, and latent transforming growth factor binding protein 1 (LTBP1) as top candidates for POGLUT2/3 substrates, as they had the greatest number of EGFs containing the putative consensus sequence [6]. Mass spectral glycoproteomic site mapping showed that more than half of their EGF repeats on these proteins are modified with an *O*-glucose monosaccharide, most at high stoichiometry (Fig. 2) [4]. This *O*-glucose modification was found on endogenous FBN1 immunopurified from human fibroblasts, recombinant proteins expressed in HEK293T cells, as well as endogenous protein isolated from mouse tissue [4,55]. Other extracellular matrix (ECM) proteins, including fibulins 2 and 5 (FBLN2, 5) and nidogen 1 (NID1) also had *O*-glucose modifications on EGFs consistent with POGLUT2/3 activity [55]. While most mapped *O*-glucosylated sites adhered to the putative consensus, some did not precisely match, with Y residues being common enough in the F position to necessitate a revision of the consensus to: C^3^-x-N-T-x-G-S-[F/Y]-x-C^4^ (Fig. 2) [4]. The conserved residues in this revised consensus were not absolute as other amino acids infrequently appeared at the conserved positions, raising the question of their requirement for substrate recognition by POGLUT2/3. Additional studies discussed below (Section 5) led to the more relaxed consensus C^3^-x-x-x-x-x-S-x-x-C^4^ which more accurately predicts mapped *O*-glucosylation sites in FBN1, FBN2, and LTBP1.

## 3. Structure and function of FBN1, FBN2, and LTBP1

FBN1, FBN2, and LTBP1 have been extensively researched for decades [57–59], and are essential for mammalian development and organ function. Fibrillins are secreted from cells, where they associate *via* N- and C-termini to form microfibrils and are incorporated into the ECM [57,58,60,61]. FBN1 microfibrils form a “beads on a string” structure, with individual units of the structure composed of 8 FBN1 monomers [61]. Tropoelastin, the soluble precursor to elastin, is secreted from cells and crosslinked to fibrillin microfibrils by lysyl oxidase, forming elastic microfibrils [62]. This creates an essential component of elastic tissue that is necessary for proper function of organs like the lungs and heart [63]. Fibrillin microfibrils in non-elastic tissues such as in the eyes and skeleton lack elastin and provide structure/flexibility and modulate signaling [64]. FBN2 is primarily expressed during embryogenesis as elastic fibers are constructed, while FBN1 expressed during embryogenesis and postnatally maintains the overall structure of these fibers [65,66].

EGFs are the most prevalent domain in the fibrillins. FBN1 features 47 EGFs, 43 of which also coordinate calcium binding (cbEGFs) (Fig. 2) [23]. The N and F/Y residues of the calcium binding consensus fall within the POGLUT2/3 consensus (Fig. 1). Calcium coordination affords increased stability between adjacent cbEGFs, which makes the linkage between the cbEGFs fairly linear and helps to stabilize the EGF fold [67–69]. Aspartic acid (D) or Asparagine (N) residues located within the POGLUT2/3 consensus can be modified by the enzyme aspartate/asparagine-β-hydroxylase (AspH), which catalyzes the addition of a hydroxyl group to the beta carbon of the D or N [70] (Fig. 1).

The LTBP proteins share EGF-like domains with the fibrillins and like the fibrillins have a number of EGF domains that are *O*-glucosylated by POGLUT2/3 [50]. LTBP1 is one of four LTBP proteins that play a crucial role in regulating TGFβ signaling [71]. LTBP1 anchors the inactive TGFβ Large Latent Complex (LLC) to fibrillin microfibrils [72]. Active TGFβ ligand can be released from the LLC either by proteases or through a pulling force generated by LTBP1 interaction with integrins on the cell surface [73].

## 4. Some Marfan syndrome FBN1 variants display aberrant elongation of POGLUT2/3 *O*-glucosylation sites

In humans, Marfan Syndrome (MFS) is caused by mutations in *FBN1* leading to deletions of segments of the protein or variation of individual amino acids [56,74]. These mutations lead to clinical features like aortic dissection, emphysema, elongated bones and misshapen rib cages, and dislocation of the lens of the eye (ectopia lentis) [75–77]. Other connective tissue disorders like Weill-Marchesani syndrome (WMS) and acromelic dysplasia are also caused by different mutations in *FBN1*, though they can have different features than MFS. This includes short stature and brachydactyly in WMS and severe short stature in acromelic dysplasia [78]. MFS and WMS are autosomal dominant diseases [74,75]. Several mouse *Fbn1* mutant models were used to understand phenotypic parallels between disease-associated mutations and artificial mutation [75]. When homozygous, the *Fbn1* mutations significantly reduce FBN1 levels and disrupt elastic fiber formation, and mutants exhibit some overlap with Marfan cardiopulmonary, ocular, and skeletal defects in animals homozygous for the mutation [75].

There are a wide variety of *FBN1* mutations that are associated with MFS. This includes deletions, frameshifts, and missense mutations. The majority of missense mutations lead to mainly loss of or addition to a cysteine, while other mutations produce variants within POGLUT2/3 sites. With POGLUT2/3 previously being described as modifying a putative, strict consensus sequence (C^3^-x-N-T-x-G-S-F/Y-x-C^4^) in folded EGF repeats, it was hypothesized that any deviation from the conserved residues in the consensus would prevent the glycosyltransferases from recognizing a site, thus preventing *O*-glucosylation.

To examine the requirement of conserved residues in the putative consensus sequence for POGLUT2/3 modification of EGFs, an alanine scan of FBN1 EGF15 was utilized in conjunction with LC-MS/MS to characterize the effect of alanine variants on glycosylation [24]. This experiment demonstrated that only the conserved serine residue between the C^3^ and C^4^ of EGF15 was absolutely required for *O*-glucosylation. Variation of other residues to alanine still allowed for modification, though in some cases the relative abundance of *O*-glucose modified peptide ions was significantly reduced. Surprisingly, in some conserved C and N variants the *O*-linked glucose was elongated with other sugars (Fig. 3). N forms part of a calcium binding consensus (Fig. 1). The elongated sugars included a Xylose-Xylose-Glucose trisaccharide as well as Sialic Acid-Galactose-Glucose (sialyllactose) trisaccharide. Elongation of the *O*-glucose monosaccharide had not previously been reported on WT FBN1, FBN2, or LTBP1.

To determine which enzymes facilitated the aberrant elongation, the N-terminal FBN1 fragment (EGFs 1–26) with N1049A substitution in EGF15 was overexpressed in glycosyltransferase knockout HEK293T backgrounds and subsequently characterized by MS/MS. Candidates for elongating the *O*-glucose were GXYLT1/2 and XXYLT1, as these enzymes were known to add two xyloses to POGLUT1-mediated *O*-glucose sites on NOTCH1 (Fig. 1) [29,30]. Alternatively, galactosyltransferase (B4GALT1) was recently shown to elongate POGLUT1-mediated *O*-glucose with a galactose, which in turn is subsequently capped by a Sialic Acid on EGF10 of NOTCH1 [26]. Expression of the NOTCH1 N1049A variant in *GXYLT1/2* double knockout (*DKO*) HEK293T cells eliminated the xylose trisaccharide, and expression in *B4GALT1 KO* HEK293T cells blocked elongation to the Sialic Acid-Galactose-Glucose trisaccharide elongation species, demonstrating that these glycosyltransferases are responsible for the aberrant elongation of *O*-glucose [24] (Fig. 3).

Several MFS related variants falling within the of POGLUT2/3 consensus also cause aberrant elongation of *O*-glucose modification sites. In general, MFS variants of conserved cysteine residues or the N (asparagine) and F/Y (phenylalanine/tyrosine) calcium binding consensus residues (C^3^-x-N-T-x-G-S-F/Y-x-C^4^) led to elongation of the *O*-glucose, whereas MFS variants of other conserved residues displayed either a reduction of *O*-glucose modification or no change at all [24]. Disrupted disulfide bonding (cysteine variants) or EGF domain stability (calcium binding variants) could impact the localized fold of an individual EGF, removing its protective status against aberrant elongation of the *O*-glucose. While the larger FBN1 protein structure may remain folded (and thus allow POGLUT2/3 recognition), these individually destabilized and elongated sites have the potential to contribute to MFS related pathological features.

## 5. Updating the potential POGLUT2/3 substrates

Based on results of the alanine scan of the POGLUT2/3 consensus, only the C residues (necessary for the characteristic EGF structure) and the S required for *O*-glucosylation were required for POGLUT2/3-mediated *O*-glucosylation of and EGF [24]. Potential new substrates excluded by the strict original consensus were identified by searching the UNIPROT database for EGF containing proteins with the redefined consensus sequence of C^3^-x-x-x-x-x-S-x-x-C^4^ (Supporting Table S1). A similar search using the original, strict consensus sequence identified 56 proteins, while using the newly revised sequence identified 101 proteins [24]. Half of the proteins identified in the search have disease causal mutations in their genes recorded in the literature (as included within their UNIPROT entries in Supporting Table S1). Site mapping of new POGLUT2/3 substrates to establish they are modified and whether disease causing mutations interfere with *O*-glucosylation. Even with the expansion of the potential POGLUT2/3 substrates, FBN1, FBN2, and LTBP1 continue to have the most POGLUT2/3 modification sites, raising the possibility that FBN1, FBN2, and LTBPs are the major biological substrates of POGLUT2/3. Notably, the revised relaxed consensus also accurately predicted all known modified EGFs on FBN1, FBN2, and LTBPs [24]. So far, POGLUT2/3 have only been demonstrated to modify EGFs. However, their ability to modify FBN1 cysteine variants [24], albeit inefficiently, raises the possibility that POGLUT2/3 could modify as yet to be identified targets.

## 6. POGLUT2/3 are essential for mouse development

POGLUT2/3 are biologically relevant transferases as demonstrated by characterization of the mouse *Poglut2* and *Poglut3* double knockout (*Poglut2/3 DKO*) [55]. Individual knockout of either glycosyltransferase resulted in viable and fertile mice with no obvious developmental defects [55]. This agreed with results from cell-based studies that showed partial biochemical redundancy of the transferases, suggesting that these enzymes could compensate each other [4,6]. When both glycosyltransferases were knocked out, the majority of mice died soon after birth. Defects observed in *Poglut2/3* DKO mice include small size, digits 2–3 syndactyly (fused digits), and missing eye or small or absent pupil at embryonic day (E) 18.5 [55].

Overall, the *Poglut2/3 DKO* reduced levels of FBN1, FBN2, and LTBP1 in lung tissues with the greatest effects on the fibrillins [55]. Interestingly, levels of FBN1 and FBN2 secreted from cultured *Poglut2/3 DKO* dermal fibroblasts was modestly reduced (30% and 50%, respectively) compared to controls, but were markedly reduced in the ECM (80%), suggesting that the *O*-glucose modification not only promotes efficient secretion but also could facilitate microfibril assembly or stability in the ECM [55,79]. Consistent with a role for fibrillin in elastic fiber formation, the levels of elastin, an essential component of elastic fibers that make up elastic connective tissue, were also reduced in *Poglut2/3 DKOs* [55]. Since, the lack of *O*-glucose on FBN1 in *Poglut2/3 DKOs* did not significantly alter the linear bead-on-a-string organization of the fibrillin microfibrils, but did subtly alter the periodicity of the fibers in the *Poglut2/3 DKO* we hypothesize that the *O*-glucose is not required for coordination of calcium, and more likely influences physical and/or functional properties of the fibrillin microfibrils [79].

We predict that neonatal lethality in the *Poglut2/3 DKOs* likely results from cardiopulmonary abnormalities related to defects in elastic fibers. Several mouse *Fbn1* mutations have defects in elastic fibers with congenital abnormalities in the lung, heart, and/or vessels with susceptibility to aortic aneurysm and neonatal death [75,76,80–83]. In addition, since TGFβ signaling was upregulated in diseased aorta in the *Fbn1^mgR/mgR^* MFS mouse model [84], this raises the question as to whether the observed altered periodicity of the fibrillin microfibrils in the *Poglut2/3 DKO* impacts TGFβ signaling [79]. Defects in fibrillin microfibrils function could also contribute to the small size and eye abnormalities observed of the *Poglut2/3 DKOs* as small stature is observed in human patients with WMS [78] and mouse *Fbn1* mutants have ocular defects [64].

Syndactyly in the *Poglut2/3 DKO* likely results from defects in FBN2 function as *Fbn2* mutant mice also have fused digits [85]. In the context of digit separation, the *O*-glucose appears to be important for reorganization of the FBN microfibrils during interdigital tongue invagination [79]. In addition, during regression of the interdigital mesenchyme by apoptosis, POGLUT2/3 mediated *O*-glucosylation is critical for modulating levels of bone-morphogenetic growth factor (BMP) and/or its inhibitor gremlin (GREM1) [79]. The unusual sensitivity of digits 2–3 to syndactyly likely stems from an earlier effect of the mutation on BMP signaling when digit periodicity is established [79].

It is also possible that altered function of other POGLUT2/3 substrates contributes to abnormalities in the *Poglut2/3 DKO*. Several of these proteins including the LTBPs and fibulins facilitate fibrillin microfibril and elastic fiber assembly and/or growth factor bio-availabilty [65,71]. *Ltbp1* knockouts die soon after birth with defects in the great vessels, valves, and heart septum [86–89]. Future studies are needed to determine whether the *Poglut2/3 DKO* similarly affects cardiac development. In humans, mutations in *LTBP4* causes Cutix Laxa, Autosomal Recessive, type IC (ARCL1C; OMIM #613177) with defects in elastogenesis that affect heart, lungs, and skin [90]. LTBP4 facilitates elastic fiber formation by promoting incorporation of FBLN5 (an essential component for elastogenesis) into fibrillin microfibrils [83,91]. Similar defects in elastic fibers are observed mouse *Ltbp4*−/− which die between postnatal day 8 (P8) and P14. The thickened aorta in *Ltbp4*−/− mutants and enlarged airspaces in *Ltbp4*−/− and *Fbln5*−/− mutants [80,83,92,93] are reminiscent of defects observed in *Poglut2/3 DKO* (Neupane et al., In Revision, *Dev Dyn*). Further characterization of *O*-glucose modifications in these substrates and the impact the sugar has on their functional interactions could be useful in identifying causation at the protein level and help to reconcile the collection of shared features the mouse models present.

## 7. Conclusions

Decades of research have demonstrated the importance of POGLUT1 and POFUT1 *O*-glycosylation in the context of NOTCH related signaling and human health and disease [16,25,26,28,42]. The discovery of POGLUT2/3 mediated *O*-glucosylation on fibrillins and related ECM proteins raises the possibility that *O*-glycans will also be relevant to the pathology of connective tissue disorders. *Poglut2/3* DKO mice partially phenocopy *Fbn1, Fbn2* and *Ltbp1* KO mice, suggesting that the *O*-glucose modification is essential for the function of these proteins. Future studies that focus on the effects of aberrant glycosylation (loss of *O*-glucose or elongation) in the context of FBN1 interactions with binding partners, microfibril incorporation, proteolytic sensitivity, and folding stability could be key in tying together clinical features of fibrillinopathies with protein functions that are influenced by glycosylation. The recently redefined POGLUT2/3 consensus sequence identified potential new substrates and sites of *O*-glucose modification. Identification of *O*-glucose site occupancy on new targets outside of the ECM would further extend our knowledge base on the possibility of *O*-glucose functions. While fibrillins and LTBPs continue to have the greatest number predicted *O*-glucosylated EGF, smaller proteins with fewer modified EGFs could also be significantly impacted by elimination or elongation of *O*-glucosylation if the *O*-glucose modulates efficiency of protein trafficking or protein-protein interactions critical for their extracellular function. With half of the newly identified proteins being associated with a disorder, this post-translational modification could play a key role in pathology for disorders that were not previously linked to glycobiology.

## Supplementary Material

1

Supplementary data to this article can be found online at https://doi.org/10.1016/j.bbagen.2026.130942.

## Figures and Tables

**Fig. 1.**
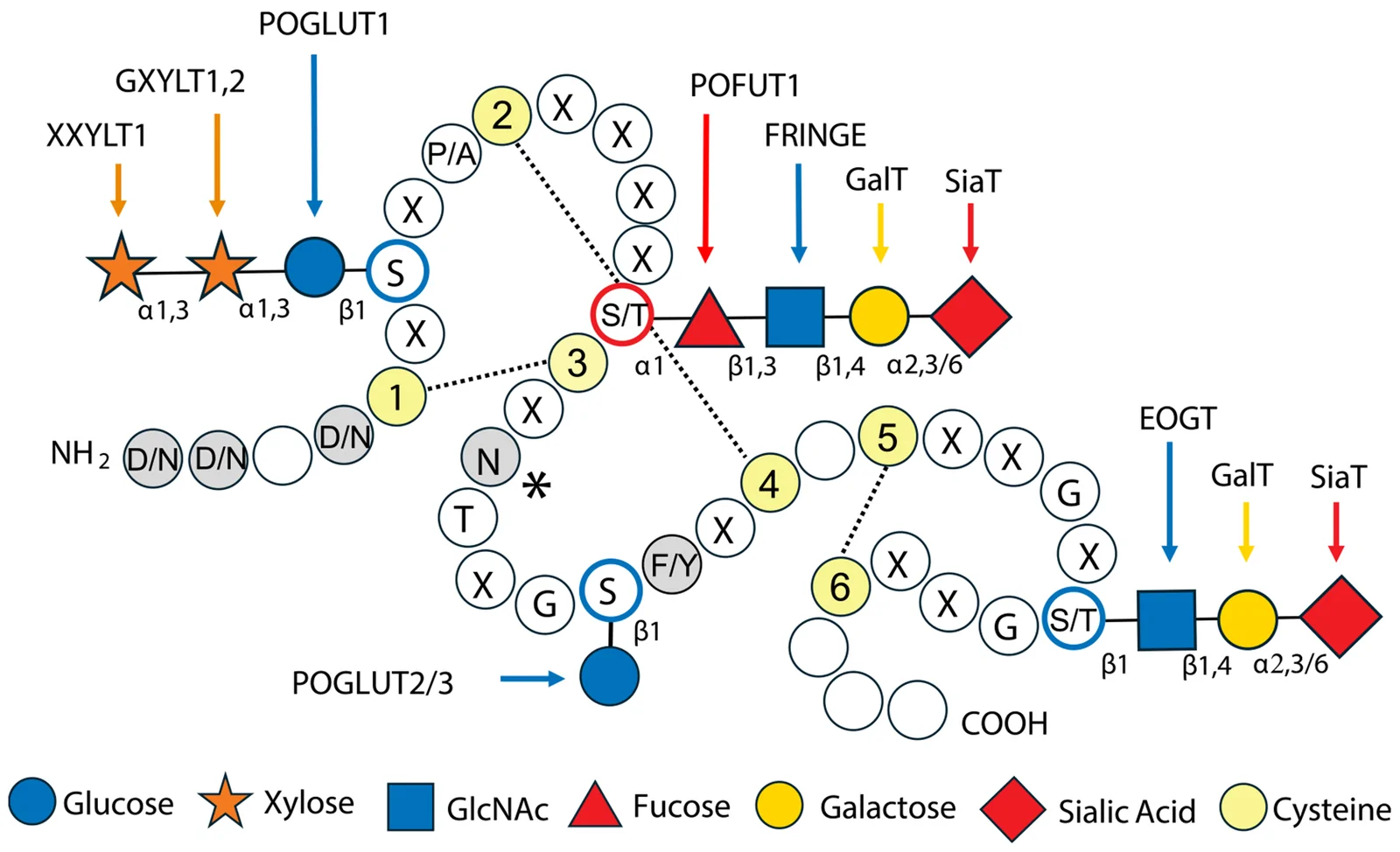
Schematic of EGF with all known *O*-glycosyltransferase modification sites and consensus sequences. The EGF consists of approximately 40–50 amino acids (string of circles) with three characteristic disulfide bonds (dotted lines) connecting cysteine residues (numbered yellow circles). POGLUT1 adds a β-linked *O*-glucose to a serine residue located between the first and second cysteines (C^1^ and C^2^, blue outlined circle). The *O*-glucose can subsequently be elongated with xyloses by GXYLT1,2 and XXYLT1. POFUT1 adds an α-linked fucose to either serine or threonine residues (S/T, red outlined circle) located between C^2^ and C^3^. The *O*-fucose can be elongated by the Fringe family of glycosyltransferases with a GlcNAc, and even further elongated by GalTs and SiaT with galactose and sialic acid, respectively. POGLUT2/3 add a β-linked *O*-glucose monosaccharide to a serine between C^3^ and C^4^ (blue outlined circle). The consensus shown is the old consensus [4] and also highlights residues required for coordination of calcium (grey circles) [23] and a beta-hydroxylation site (*). The new consensus is C^3^-x-x-x-x-x-S-x-x-C^4^ [24]. The *O*-glucose is not normally extended except in certain cases where the original sequence is altered [24]. EOGT adds a β-linked GlcNAc to serine or threonine residues between C^5^ and C^6^ (blue outlined circle). The *O*-GlcNAc can be elongated with galactose and sialic acid. Symbol nomenclature for glycans represent all known sugar modifications across EGFs. Modified from [4,23–26] with permission. (For interpretation of the references to colour in this figure legend, the reader is referred to the web version of this article.)

**Fig. 2.**
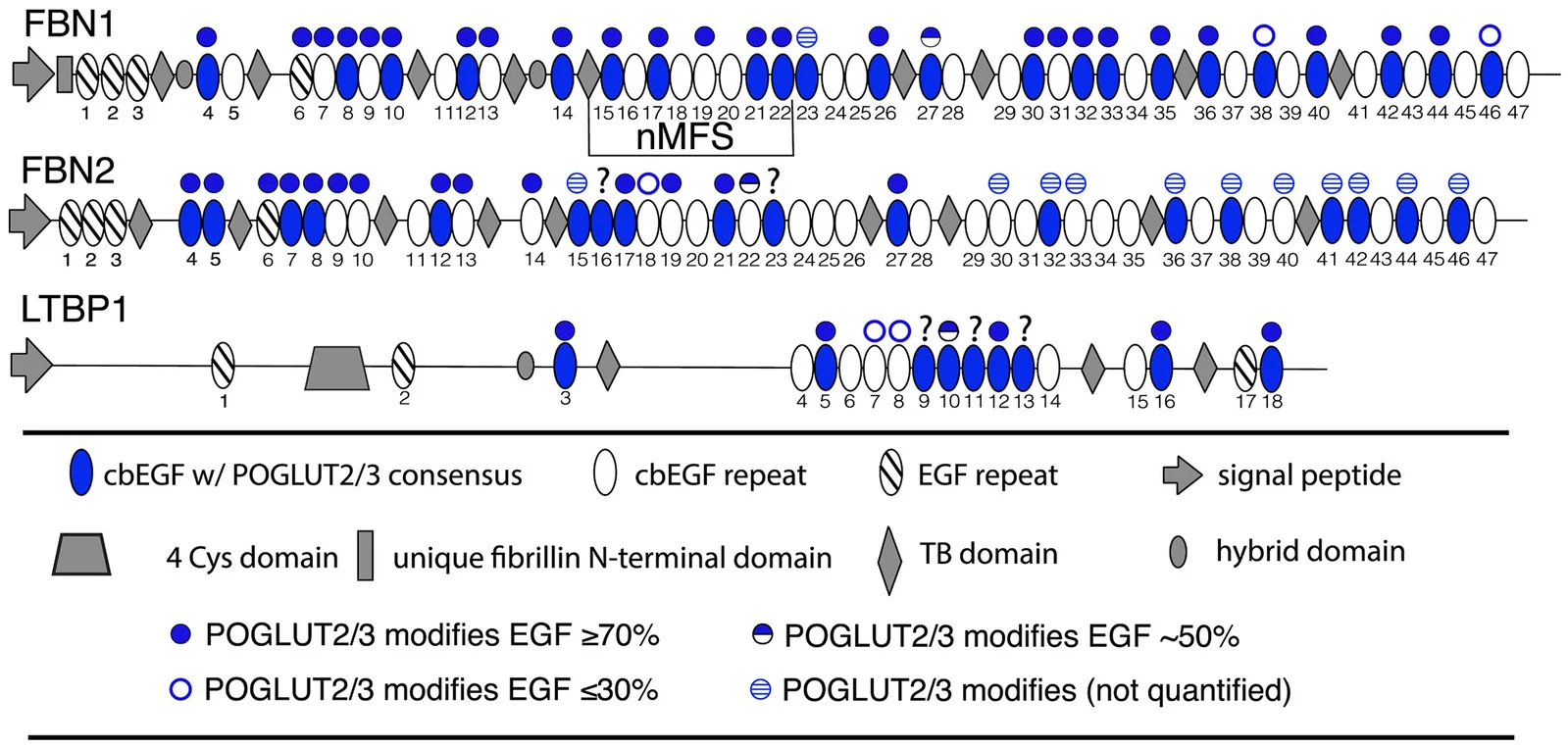
Distribution of POGLUT2/3-medated *O*-glucosylation on FBN1, FBN2, and LTBP1 EGF. Comparison of the domain structure, consensus site distribution, and *O*-glucosylation levels on FBN1, FBN2, and LTBP1. FBN1 and FBN2 have similar domain organization with the exception that FBN2 lacks a hybrid domain next to EGF14 and the unique fibrillin N-terminal domain. nMFS refers to the neonatal region of FBN1, where severe MFS clinical features are most associated [56]. LTBP1 shares multiple domains with the fibrillins. EGFs predicted to be modified by POGLUT2/3 using the old consensus sequence are indicated by blue filled ovals. Summary of semiquantitative mass spectral analyses of *O*-glucosylated peptides are indicated above the EGF domains by blue circles with varying shading. Refer to the key below for % modification. “?” denotes EGFs with a consensus site where *O*-glucosylation has not yet been confirmed [4]. Abbreviations: calcium binding EGFs (cbEGF), non-calcium binding EGFs (EGF), and TGFβ binding protein-like (TB) domains. LTBP1 is smaller overall and contains fewer EGFs. All three proteins are *O*-glucosylated by POGLUT2/3. Reproduced from [4] with minor modifications with permission. (For interpretation of the references to colour in this figure legend, the reader is referred to the web version of this article.)

**Fig. 3.**
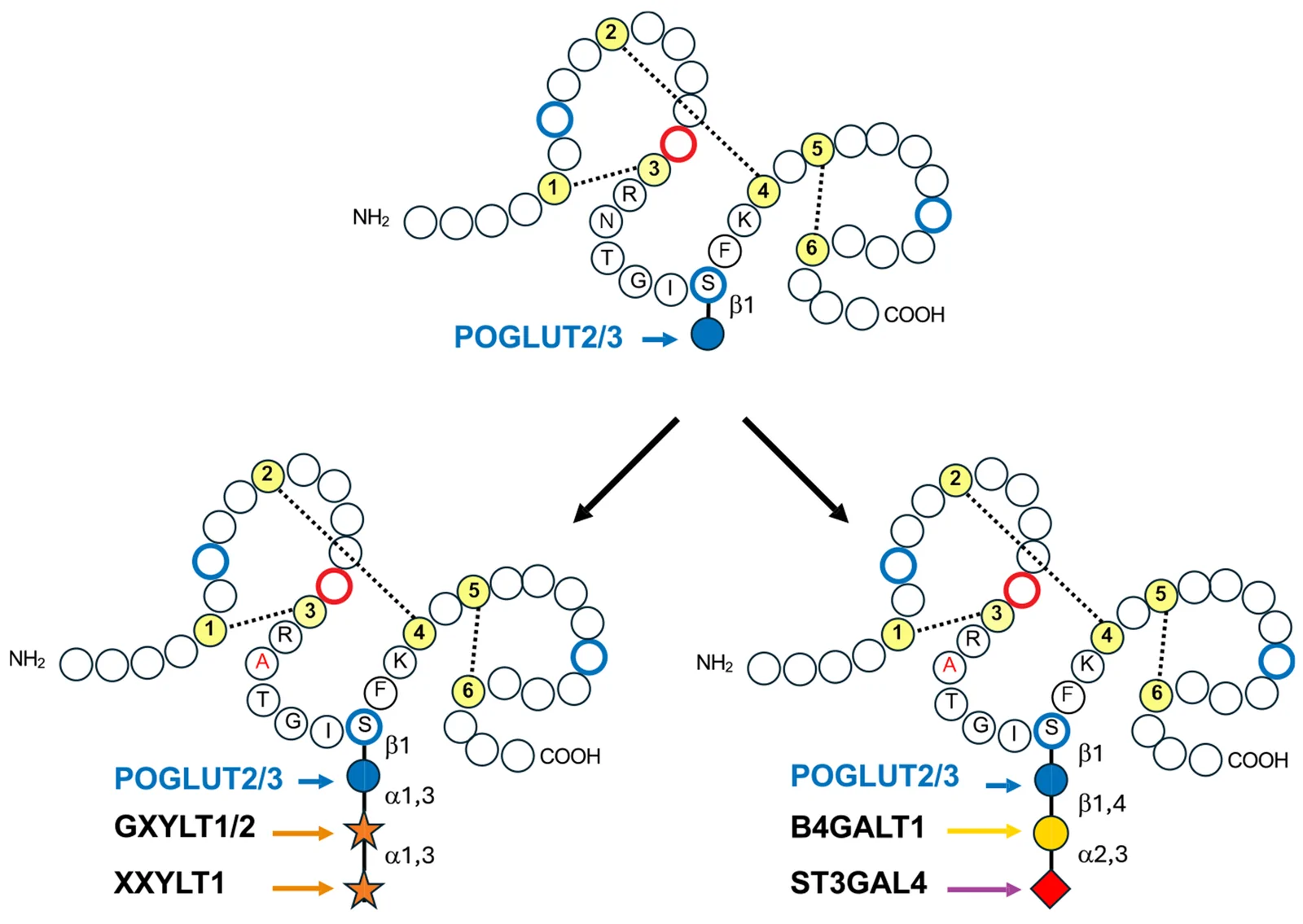
POGLUT2 and POGLUT3 sites can undergo aberrant elongation. (Above) Cartoon representation of FBN1 EGF15 with original consensus sequence. Coloring is same as in Fig. 1. (Below) Two distinct elongation pathways when the N is mutated to an alanine (A, red) [24]. Mutations disrupting calcium binding or disulfide bonds have the same effect. Reproduced from [24] with minor modifications. (For interpretation of the references to colour in this figure legend, the reader is referred to the web version of this article.)

## Data Availability

This is a review article. The data is in the papers referenced.
